# Risk Factors Associated With Unplanned Conversion to Open in Radical and Partial Nephrectomy

**DOI:** 10.7759/cureus.85168

**Published:** 2025-06-01

**Authors:** Benjamin A Fink, Young Son, Kimberly C Toumazos, Virgil K DeMario, Joseph P Flemming, Nikki Covelle, Logan B Wesemann, Edward Wu, Vaishnavi J Patel, Devki Patel, Thomas J Mueller

**Affiliations:** 1 Department of Urology, Jefferson Health, Stratford, USA; 2 Department of Urology, Jefferson Stratford Hospital, Stratford, USA; 3 Department of Urology, University of Kentucky College of Medicine, Lexington, USA; 4 Department of Clinical and Applied Science Education, University of the Incarnate Word School of Osteopathic Medicine, San Antonio, USA; 5 Department of Medicine, Rowan-Virtua School of Osteopathic Medicine, Stratford, USA; 6 Department of Urology, Rocky Vista University College of Osteopathic Medicine, Ivins, USA; 7 Department of Urology, Maimonides Medical Center, Brooklyn, USA; 8 Office of Research and Innovation, University of the Incarnate Word School of Osteopathic Medicine, San Antonio, USA; 9 Office of Research and Innovation, Texas Tech University Health Sciences Center Paul L. Foster School of Medicine, Lubbock, USA

**Keywords:** minimally invasive surgery, partial nephrectomy, radical nephrectomy, renal cell carcinoma, unplanned conversion to open

## Abstract

Introduction

Minimally invasive surgery (MIS), such as laparoscopic and robotic techniques, has become standard in kidney surgery due to its benefits in offering faster recovery while maintaining comparable oncologic outcomes when compared to open surgery. However, some cases still require unexpected conversion to open surgery, which can increase the risk of postoperative complications and increase the 30-day readmission risk. Predictors and risk factors for these conversions remain underexplored. This study uses the American College of Surgeons National Surgical Quality Improvement Program (ACS NSQIP) database to identify predictors and outcomes in nephrectomy patients, therefore improving patient selection for open surgeries.

Methods

Using the 2019-2020 NSQIP data, this retrospective study analyzed 14,186 patients, 4,862 of whom met the inclusion criteria of nephrectomy for kidney cancer. Patients were subcategorized into planned MIS (n=4,756) and unplanned conversion to open (UCO) (n=106). The Wilcoxon signed-rank test was utilized for continuous variables, and Fisher’s exact test was performed for categorical variables, including chi-squared analysis. Statistical significance was set at a P-value of <0.05.

Results

Unplanned conversion to open rate was 2.2%. There was an increased incidence of unplanned conversion to open in T3 and T4 tumors (30.2% and 8.5%) compared to the planned group (21.2% and 0.50%). Patients in the American Society of Anesthesiologists (ASA) classification 3 and 4 categories were more likely to be within the unplanned group. Outcomes (readmission, blood transfusion, mean operative time, the length of stay, prolonged nil per os/nasogastric tube {NPO/NGT}, and lymphocele/lymphatic leak) were compared between the groups.

Conclusion

Higher tumor stage, higher ASA classification, deranged kidney function, and raised prothrombin time/international normalized ratio (PT/INR) were predictive factors for unplanned conversions, with worse outcomes such as prolonged hospitalizations and postoperative complications.

## Introduction

Renal cell carcinoma (RCC), ranking as the seventh most prevalent neoplasm worldwide, poses a significant challenge necessitating meticulous surgical intervention, with the choice between partial nephrectomy (PN) and radical nephrectomy (RN) being crucial [1-4]. Minimally invasive surgery (MIS), including laparoscopic and robotic techniques, is preferred for PN and RN due to shorter hospital stays, less pain, faster recovery, and strong oncologic outcomes [4-8].

Despite the growing preference for MIS among both patients and surgeons, its intrinsic limitations and unforeseen intraoperative complications may necessitate an unplanned conversion to open (UCO). Recent studies have documented UCO rates in nephrectomy ranging from 2% to 6% [9-12]. Although previous studies have identified risk factors for UCO in minimally invasive PN, such as obesity and surgical history, there remains a knowledge gap regarding UCO risk factors for higher-stage disease [13,14]. Moreover, conflicting evidence surrounds the impact of UCO on postoperative outcomes [4,10].

In pursuit of greater clarity, our study explores factors associated with UCO in nephrectomy for kidney cancer. Leveraging data from the American College of Surgeons National Surgical Quality Improvement Program (ACS NSQIP), we compared preoperative with intraoperative risk factors between those undergoing MIS and UCO. We also analyzed postoperative outcomes for both cohorts to clarify the effects of UCO by comparing results with planned open PN or RN. We hypothesized that our findings would align with earlier studies, substantiating independent predictors of UCO in the context of nephrectomy.

## Materials and methods

Data source

The ACS NSQIP is a Health Insurance Portability and Accountability Act (HIPAA)-compliant data file that includes nephrectomy data from 6,585 cases in 2020 (122 hospitals) and 7,818 cases in 2019 (117 hospitals) in the United States. The ACS NSQIP is de-identified and nationally available, exempting the study from institutional review board approval.

Study population and aim

The 2019-2020 ACS NSQIP nephrectomy database was retrospectively reviewed for patients who underwent radical or partial nephrectomy for malignant etiologies. Postoperative diagnosis to determine the inclusion criteria used included the following: malignant carcinoid tumor of the kidney; malignant neoplasm of connective and soft tissue; unspecified, malignant neoplasm of the kidney, except the renal pelvis; malignant neoplasm of the left kidney, except the renal pelvis; malignant neoplasm of the right kidney, except the renal pelvis; malignant neoplasm of unspecified kidney, except renal pelvis; neoplasm of uncertain behavior of the left kidney; and personal history of other malignant neoplasm of the kidney. The data were then subcategorized into two groups: planned minimally invasive surgery (MIS)-only group (including laparoscopic, endoscopic, robotic, and other MIS) (n=4,756) and unplanned conversion to an open from robotic, laparoscopic, or endoscopic procedure (n=106). Radical and partial nephrectomy were determined by Current Procedural Terminology (CPT) codes, which can be seen in the Appendices.

Preoperative and intraoperative factors for UCO nephrectomy for kidney cancer were analyzed as a primary endpoint (Table 1). A secondary aim was to determine the postoperative outcomes (Table 2) associated with the UCO and the planned MIS groups.

**Table 1:** Patient Characteristics: Unplanned Conversion Versus Minimally Invasive Surgery P-value represents the t-test for continuous variables and chi-squared for categorical variables ASA, American Society of Anesthesiologists; BUN, blood urea nitrogen; PTT, partial thromboplastin time; INR, international normalized ratio; COPD, chronic obstructive pulmonary disorder; TNM, tumor, node, metastasis

	Total Cohort	Unplanned Conversion to Open	Minimally Invasive Surgery	P-value
	n=4,862	n=106 (2.2%)	n=4,756 (97.8%)	
Patient Demographics				
Mean Age (Range)	61.28 (19-89)	62.58 (32-88)	61.25 (19-89)	0.265
Male Gender (%)	3,170	72 (67.92%)	3,098 (65.14%)	0.622
Non-Caucasian Race (%)	1,580 (32.50%)	39 (36.79%)	1,541 (32.40%)	0.395
Hispanic Ethnicity (%)	353	4 (3.77%)	349 (7.34%)	0.226
Past Medical History/Comorbidities				
Admitted From Other Than Home (%)	23	2 (1.9%)	21 (0.4%)	0.171
Preoperative Independent Health Status (%)	40 (0.82%)	0 (0.0%)	40 (0.8%)	0.686
>10% Body Weight Decrease in Six Months (%)	66	4 (3.8%)	62 (1.3%)	0.055
Preoperative Mechanical Bowel Preparation (%)	754 (15.5%)	11 (10.4%)	743 (15.6%)	0.180
Preoperative Oral Antibiotic Preparation (%)	70	1 (0.9%)	69 (1.5%)	1.000
Prior Pelvic Surgery (%)	1,608 (33.1%)	41 (38.7%)	1,567 (32.9%)	0.256
Prior Pelvic Radiotherapy (%)	75	0 (0.0%)	75 (1.6%)	0.413
Chemotherapy (Within 90 Days of Surgery) (%)	103 (2.1%)	3 (2.8%)	100 (2.1%)	0.492
Transfusion Within 72 Hours of Surgery (%)	15	2 (1.9%)	13 (0.3%)	<0.001
Currently on Hemodialysis (%)	124 (2.6%)	8 (7.5%)	116 (2.4%)	0.003
Current Smoker (Within the Past Year) (%)	789	13 (12.3%)	776 (16.3%)	0.324
Diabetes Mellitus Treated With Insulin (%)	342 (7.0%)	8 (7.5%)	334 (7.0%)	0.987
Diabetes Mellitus Treated With Oral Medication	754	18 (3.8%)	736 (15.5%)	0.773
Disseminated Cancer (%)	155 (3.2%)	10 (9.4%)	145 (3.0%)	<0.001
History of Severe COPD (%)	184	2 (1.9%)	182 (3.8%)	0.437
Hypertension (Treated With Medication) (%)	2,984 (61.4%)	69 (65.1%)	2,915 (61.3%)	0.487
Steroid Use for Chronic Medical Condition (%)	195	5 (4.7%)	190 (4.0%)	0.901
Dyspnea (%)	285 (5.9%)	12 (11.3%)	273 (5.8%)	0.027
Bleeding Disorder (%)	124	6 (5.7%)	118 (2.55)	0.081
ASA Classification				
Class 1 (%)	62	1 (0.9%)	61 (1.3%)	1.000
Class 2 (%)	1,792 (36.9%)	25 (23.6%)	1,767 (37.2%)	0.006
Class 3 (%)	2,834	71 (67.0%)	2,763 (58.1%)	0.083
Class 4 (%)	171 (3.5%)	8 (7.5%)	163 (0.04%)	0.044
TNM Classification				
T1, T1a, T16 (%)	3,481 (71.6%)	57 (53.8%)	3,424 (72.0%)	<0.001
T2, T2a, T2b (%)	306	8 (7.5%)	298 (6.3%)	0.738
Т3, Т3а, T3b, Т3С (%)	1,042 (21.4%)	32 (30.2%)	1,010 (21.2%)	0.036
T4 (%)	33	9 (8.5%)	24 (0.2%)	<0.001
Nx (%)	4,199 (86.4%)	74 (69.8%)	4,125 (86.7%)	<0.001
NO (%)	472	21 (19.8%)	451 (9.5%)	<0.001
N1 (%)	61 (1.3%)	4 (3.8%)	57 (1.2%)	0.043
MO (%)	2,196	44 (41.5%)	2,152 (45.2%)	0.505
M1 (%)	67 (1.4%)	5 (4.7%)	62 (1.3%)	0.015
Preoperative Laboratory Values				
Elevated Creatinine (≥1.6 mg/dL) (%)	337 (6.9%)	15 (14.2%)	322 (6.8%)	0.004
Elevated BUN (≥21 mg/dL) (%)	1,155	33 (31.1%)	1,122 (23.6%)	0.083
Elevated PTT (≥36 seconds) (%)	233 (4.8%)	13 (12.3%)	220 (4.6%)	0.001
Elevated INR (≥1.16) (%)	271	12 (11.3%)	259 (5.5%)	0.039
Decreased Platelet Count (≤149 × 10%/L) (%)	567 (11.7%)	17 (16.0%)	550 (11.6%)	0.063
Nephrectomy				
Partial Nephrectomy	2,568 (52.8%)	25 (23.6%)	2,543 (53.5%)	<0.001
Radical Nephrectomy	2,294	81 (76.4%)	2,213 (46.5%)	<0.001

**Table 2:** Postoperative Outcome Frequency Table: Unplanned Conversion to Open Versus Minimally Invasive Surgery P-value represents the t-test for continuous variables and chi-squared for categorical variables CVA, cerebrovascular accident; CPR, cardiopulmonary resuscitation; DVT, deep vein thrombosis; NPO, nil per os; NGT, nasogastric tube

Outcome Variables	Total Cohort	Unplanned Conversion to Open	Minimally Invasive Surgery	P-value
	n=4,862	n=106 (2.2%)	n=4,756 (97.8%)	
Readmission to Hospital (%)	227 (4.7%)	17 (16.0%)	210 (4.4%)	<0.001
Mean Total Operating Time in Minutes (Range)	176.42 (4-769)	255.08 (66-732)	174.67 (4-769)	<0.001
Mean Length of Hospital Stay (Range)	2.37 (0-36)	5.07 (1-27)	2.31 (0-36)	<0.001
Mean Days From Operation to Discharge (Range)	2.28 (0-30)	4.76 (1-21)	2.22 (0-30)	<0.001
Surgical Site Infection (SSI)				
Superficial Incisional SSI (%)	62 (1.3%)	3 (2.8%)	59 (1.2%)	0.315
Deep Incisional SSI (%)	7 (0.1%)	2 (1.9%)	5 (0.1%)	<0.001
Organ Space SSI (%)	27 (0.6%)	6 (5.7%)	21 (0.4%)	<0.001
Postoperative Complications				
Sepsis (%)	20 (0.4%)	2 (1.9%)	18 (0.4%)	0.103
Septic Shock (%)	10 (0.2%)	1 (0.9%)	9 (0.2%)	0.541
Urinary Tract Infection (%)	52 (1.1%)	0 (0.0%)	52 (1.1%)	0.545
Acute Renal Failure (%)	15 (0.3%)	4 (3.8%)	11 (0.2%)	<0.001
Pneumonia (%)	54 (1.1%)	1 (0.9%)	53 (1.1%)	1.000
Requiring Bleeding Transfusion (%)	189 (3.9%)	35 (33.0%)	154 (3.2%)	<0.001
CVA/Stroke With Neurological Deficit (%)	6 (0.1%)	0 (0.0%)	6 (0.1%)	1.000
Cardiac Arrest Requiring CPR (%)	16 (0.3%)	1 (0.9%)	15 (0.3%)	0.796
Myocardial Infarction (%)	26 (0.5%)	1 (0.9%)	25 (0.5%)	1.000
Pulmonary Embolism (%)	24 (0.5%)	3 (2.8%)	21 (0.4%)	0.006
DVT/Thrombophlebitis (%)	26 (0.5%)	4 (3.8%)	22 (0.5%)	<0.001
Rectal Injury (%)	3 (0.1%)	0 (0.0%)	3 (0.1%)	1.000
Unplanned Intubation (%)	18 (0.4%)	0 (0.0%)	18 (0.4%)	1.000
Wound Disruption (%)	17 (0.4%)	4 (3.8%)	13 (0.3%)	<0.001
On Ventilator for >48 Hours (%)	17 (0.3%)	2 (1.95)	15 (0.3%)	0.060
*Clostridium difficile* Colitis (%)	10 (0.2%)	1 (0.9%)	9 (0.2%)	0.541
Prolonged NPO or NGT Use (%)	82 (1.7%)	13 (12.3%)	69 (1.5%)	<0.001
Lymphocele/Lymphatic Leak (%)	82 (1.7%)	6 (5.7%)	76 (1.6%)	0.005
Urinary Leak/Fistula (%)	18 (0.4%)	0 (0.0%)	18 (0.4%)	1.000
Unplanned Reoperation (%)	74 (1.5%)	8 (7.5%)	66 (1.4%)	<0.001
Mortality (%)	12 (0.2%)	1 (0.9%)	11 (0.2%)	0.232

Statistical analysis

Descriptive statistics were used to compare variables. Frequencies, proportions, and chi-squared analysis were utilized for categorical variables. For continuous variables, the mean, standard deviation, and t-test were reported. Multivariate logistic regression was performed for statistically significant preoperative/intraoperative variables to identify independent factors associated with UCO. A second multivariable logistic regression was performed for significant postoperative variables to predict the postoperative factors associated with UCO groups. Statistical analysis was conducted using R (v4.0.2) (R Foundation for Statistical Computing, Vienna, Austria), with significance set at <0.05 [15]. Postoperative complications and outcomes were assessed using the standardized and validated definitions provided by the American College of Surgeons National Surgical Quality Improvement Program (ACS NSQIP).

## Results

Minimally invasive surgery versus unplanned conversion to open

A total of 4,862 patients were included in the analysis, among which 106 (2.20%) were in the UCO group and 4,756 (97.8%) were in the MIS group. The mean age, gender, and proportion of non-Caucasian race and Hispanic ethnicity were similar between the groups. Transfusion within 72 hours of surgery was observed in two (1.9%) of UCO patients compared to 13 (0.3%) of MIS patients (P<0.001). Similarly, eight (7.5%) of UCO patients were on hemodialysis compared to 116 (2.4%) of MIS patients (P=0.003). Disseminated cancer was present in 10 (9.4%) of UCO patients and 145 (3.0%) of MIS patients (P<0.001) (Table 1). With respect to American Society of Anesthesiologists (ASA) classification, there was a higher proportion of ASA class 4 patients in the UCO group compared to the MIS group (P=0.044). Conversely, a higher proportion of ASA class 2 patients presented in the MIS group compared to the UCO group (P=0.006). The UCO group had a higher percentage of T3 (P=0.036) and T4 (P<0.001) patients, as well as N1 (P=0.043) and M1 (P=0.015) classifications. Preoperative laboratory values showed that the UCO group had a higher proportion of patients with elevated creatinine levels (P=0.004), elevated partial thromboplastin time (PTT) levels (P=0.001), and elevated international normalized ratio (INR) levels (P=0.039). The UCO group had a lower percentage of PN (P<0.001) and a higher percentage of RN (P<0.001) compared to the MIS group. Full statistical comparisons for these characteristics are detailed in Table 1.

Postoperative outcomes showed several differences between the UCO and MIS cohorts (Table 2). UCO patients had higher readmission rates (16.0% versus 4.4%, P<0.001), longer operating times (255.08 versus 174.67 minutes, P<0.001), and longer hospital stays (5.07 versus 2.31 days, P<0.001). Furthermore, the UCO group had higher rates of deep incisional surgical site infection (SSI) (P<0.001), organ space surgical site infection (P<0.001), acute renal failure (P<0.001), blood transfusion requirement (P<0.001), pulmonary embolism (P=0.006), deep vein thrombosis/thrombophlebitis (P<0.001), wound disruption (P<0.001), prolonged nil per os (NPO) or nasogastric tube (NGT) use (P<0.001), lymphocele/lymphatic leak (P=0.005), and unplanned reoperation (P<0.001). Full statistical comparisons for these characteristics are detailed in Table 2.

Multivariate regression analysis identified pathological kidney cancer staging as an independent factor that contributed to unplanned conversion to open partial or radical nephrectomy showing pT3a (OR=2.15, 95% CI: 1.03-4.38, and P=0.036) and T4 (OR=20.4, 95% CI: 5.40-70.3, and P<0.001) (Table 3). Intraoperatively, UCO had longer total operative time (OR=2.14 and 95% CI: 1.30-3.65) and higher bleeding transfusion rates (OR=5.05 and 95% CI: 3.01-8.33) compared to the MIS group (Table 4).

**Table 3:** Preoperative Risk Factors of Unplanned Conversion to Open Nephrectomy ASA, American Society of Anesthesiologists; PTT, partial thromboplastin time; INR, international normalized ratio

Preoperative Factors	Univariate Analysis		Multivariate Analysis	
	OR (95% CI)	P-value	OR (95% CI)	P-value
Elevated Creatine (>1.6 mg/dL)	2.34 (1.28-3.97)	0.003	1.15 (0.28-3.38)	0.825
Elevated PTT (>36 seconds)	2.97 (1.49-5.58)	0.001	2.20 (0.98-4.58)	0.055
Elevated INR (>1.16)	2.08 (1.04-3.83)	0.026	1.17 (0.46-2.65)	0.730
Transfusion Within 72 Hours of Surgery	7.02 (1.09-25.8)	0.011	0.93 (0.03-12.5)	0.960
Currently on Hemodialysis	3.27 (1.43-6.47)	0.002	1.44 (0.18-8.78)	0.704
Disseminated Cancer	3.31 (1.59-6.19)	<0.001	1.79 (0.60-4.33)	0.280
Dyspnea	2.10 (1.08-3.72)	0.018	1.79 (0.60-4.33)	0.280
ASA Classification				
Class 1	Reference	-	Reference	-
Class 2	0.45 (0.13-2.81)	0.279	0.14 (0.03-1.00)	0.020
Class 3	0.81 (0.25-4.99)	0.772	0.26 (0.07-1.77)	0.093
Class 4	1.55 (0.37-10.4)	0.588	0.33 (0.05-2.80)	0.260
T Malignancy Stage				
T1 (T1a, T1b)	Reference	-	Reference	-
T2 (T2a, T2b)	1.161 (0.70-3.22)	0.211	1.64 (0.38-4.93)	0.434
T3	1.51 (0.37-4.17)	0.489	2.26 (0.33-8.69)	0.304
T3a	1.78 (1.10-2.82)	0.016	2.15 (1.03-4.38)	0.036
T3b	12.9 (2.91-40.8)	<0.001	0.00 (NA)	-
T3c	0.00 (NA)	-	0.00 (NA)	-
T4	22.5 (9.55-49.1)	<0.001	20.5 (5.40-70.3)	<0.001
M Malignancy Stage				
M1	3.75 (1.29-8.66)	0.005	1.28 (0.20-5.65)	0.791

**Table 4:** Univariate and Multivariate Analysis of Postoperative Outcomes of Unplanned Conversion to Open Nephrectomy DVT, deep vein thrombosis; NPO, nil per os; NGT, nasogastric tube

Postoperative Factors	Univariate Analysis		Multivariate Analysis	
	OR (95% CI)	P-value	OR (95% CI)	P-value
Total Operating Time (Minutes)				
<139	0.64 (0.32-1.25)	0.201	0.61 (0.29-1.22)	0.168
139-193	Reference	-	Reference	-
>193	3.26 (2.04-5.41)	<0.001	2.14 (1.30-3.65)	0.004
Readmission to Hospital	4.13 (2.34-6.90)	<0.001	2.67 (1.33-5.04)	0.007
Length of Hospital Stay (>2 Days)	9.57 (6.02-15.9)	<0.001	5.61 (3.41-9.57)	<0.001
Deep Incisional Surgical Site Infection	18.3 (2.60-85.9)	<0.001	1.68 (0.07-17.6)	0.703
Organ Space Surgical Site Infection	13.5 (4.88-32.3)	<0.001	1.83 (0.49-5.99)	0.353
Acute Renal Failure	16.9 (4.63-50.4)	<0.001	4.52 (0.88-18.3)	0.068
Bleeding Transfusion	14.7 (9.45-22.6)	<0.001	5.05 (3.01-8.33)	<0.001
DVT/Thrombophlebitis	8.44 (2.44-22.5)	<0.001	0.51 (0.07-2.21)	0.396
Wound Disruption	14.3 (3.98-41.2)	<0.001	6.51 (1.03-39.0)	0.047
Prolonged NPO or NGT Use	9.50 (4.87-17.2)	<0.001	1.90 (0.77-4.29)	0.159
Lymphocele/Lymphatic Leak	3.69 (1.41-8.02)	0.003	0.57 (0.17-1.64)	0.317
Unplanned Reoperation	5.80 (2.51-11.7)	<0.001	0.58 (0.13-1.86)	0.398

Planned open nephrectomy versus unplanned conversion to open

A total of 2,183 (95.4%) underwent planned open nephrectomy, whereas 106 (4.6%) underwent UCO. The mean age was similar between the two groups, with 62.01 years for planned open and 62.58 years for UCO (P=0.610). Gender and ethnicity distributions were also similar between the two groups.

Preoperative characteristics and comorbidities were mostly similar between the planned open and UCO groups, except for a higher percentage of hemodialysis patients in the UCO group (7.55% versus 2.29%, P=0.002). ASA classification, TNM classification, and preoperative laboratory values were largely comparable (Table 3). The UCO cohort had a higher proportion of T4 stage patients (P=0.027) and elevated PTT (P=0.023). More UCO patients underwent radical nephrectomy (P<0.001), while more planned open patients had partial nephrectomy (P<0.001). Full statistical comparisons for these characteristics can be seen in Table 1.

Postoperative outcomes between the planned open and UCO cohorts showed significant differences in readmission rates, operating time, SSIs, transfusion requirements, wound disruption, prolonged NPO/NGT use, and unplanned reoperation. Readmission rates were 7.05% for planned open and 16.04% for UCO (P=0.002). The mean operating time was 182.70 minutes for planned open and 255.10 minutes for UCO (P<0.001). UCO had higher rates of deep incisional SSIs (1.89% versus 0.14%, P=0.008), organ space SSIs (5.66% versus 1.57%, P=0.006), transfusion requirements (33.02% versus 21.38%, P=0.008), wound disruption (3.77% versus 0.38%, P<0.001), prolonged NPO/NGT use (12.26% versus 5.24%, P=0.007), and unplanned reoperation (9.43% versus 2.67%, P<0.001).

On multivariate analysis, we identified several factors independently associated with UCO nephrectomy (Table 3). These included pT2 (pT2a, pT2b) (OR=0.36, 95% CI: 0.15-0.76, and P=0.012), pT3 (pT3a, pT3b, pT3c) (OR=0.18, 95% CI: 0.04-0.52, and P=0.006), pT3a (OR=0.27, 95% CI: 0.15-0.49, and P<0.001), pT3b (OR=0.10, 95% CI: 0.02-0.32, and P=0.001), and T4 malignancy stage (OR=0.26, 95% CI: 0.10-0.65, and P=0.006). Total operating time was also significantly longer for the UCO with >206 minutes (OR=2.66, 95% CI: 1.64-4.40, and P<0.001). There was no difference in postoperative bleeding transfusion (OR=1.18 and 95% CI: 0.69-4.72).

## Discussion

This retrospective cohort study investigated risk factors for unplanned conversions from minimally invasive to open surgery during partial or radical nephrectomy for RCC. Advanced pathological stages, particularly T3a and T4, were strong independent predictors. Beyond identifying risk factors, we explored the ramifications of unplanned conversions on postoperative outcomes, uncovering associations with adverse events compared to minimally invasive surgeries. A comparative analysis of planned open nephrectomy and UCO showed similar complication rates but significantly longer operating times for the UCO group (P<0.001).

The link between T3/T4 disease and UCO may involve anatomical and physiological factors such as tumor hypervascularity, friability, and invasion depth. High-grade tumors with increased vascular endothelial growth factor (VEGF) and platelet-derived growth factor (PDGF) levels can complicate surgery, often requiring open nephrectomy for better vessel visualization [16]. Although radius, exophytic/endophytic properties of the tumor, nearness of tumor to the collecting system or sinus in millimeters, anterior/posterior descriptor, and the location relative to the polar line (RENAL) nephrometry scores were unavailable, evidence suggests that higher-stage tumors hinder minimally invasive approaches by altering kidney orientation, increasing metastasis risk, and impairing organ visualization [7]. Tumors with greater depth and contiguous spread may require longer dissection, increasing ischemic duration and the risk of complications [11,17]. In advanced-stage cases with limited operative space, open surgery tends to be the preferred approach, aligning with the complexities inherent in advanced-stage RCC. In our cohort, the UCO group had a lower proportion of PN and a higher proportion of RN. Multivariate regression analysis further identified pT3a and T4 tumors as independent predictors of UCO. These results suggest that while PN and RN differ technically, the presence of advanced disease and its associated anatomical challenges may be a stronger driver of conversion risk than procedure type alone.

Additionally, our study found a higher proportion of ASA class 4 patients in the UCO group compared to the MIS group, agreeing with previous literature that patients with greater systemic disease burden may be more prone to UCO [9]. ASA class 4 indicates patients with severe systemic disease that is a constant threat to life, which may limit cardiopulmonary reserve and reduce tolerance for prolonged pneumoperitoneum or intraoperative instability [5]. These patients may require UCO in response to intraoperative hemodynamic or respiratory concerns. This is consistent with ASA’s stratification purpose, which emphasizes the increased perioperative risk in patients with higher ASA scores [5]. Our findings support the inclusion of preoperative physical status as a relevant predictor of UCO risk, aligning with previous findings that encourage this consideration during pre-surgical planning and risk stratification.

After analyzing unplanned conversions from MIS, we found higher readmission and transfusion rates in the UCO group compared to planned open nephrectomy. However, a sub-analysis adjusted for open factors showed no significant difference in readmission or bleeding risk (P=0.520 and P=0.299), suggesting that these differences are likely due to open surgery itself, rather than factors associated with unplanned conversion. UCO patients also had longer operating times, higher odds of radical nephrectomy, and similar complications to both MIS and UCO groups. Additionally, the planned open group had higher pathological staging, while UCO patients showed lower odds of advanced staging. These findings emphasize the challenges of MIS for higher-stage kidney cancer and question the accuracy of imaging in staging.

The lack of clinical staging and presence of a tumor thrombus in the NSQIP database limits the analysis of upstaging or downstaging dynamics and their correlation with UCO in RCC. Despite this, cross-sectional imaging, particularly CT (87% sensitivity and 74.5% specificity) and MRI (90% sensitivity and 75% specificity), shows promise for predicting pathological staging in RCC [18], but detection rates vary by pathological stage, with T3a renal invasion detection sensitivity ranging from 59% to 69% and T3b and T3c detection rates remaining unexplored [19]. This variability may explain higher UCO rates in T3a and T4 stages, as clinical staging often occurs intraoperatively. RCC’s complexity, with inferior vena cava (IVC) and tumor thrombus vessel wall invasion of up to 10% and 23%, respectively, adds to the challenge [17,20,21]. Despite using imaging predictors such as tumor IVC diameter and volume for IVC wall invasion, detection rates remain at 66% [22,23]. Consequently, intraoperative visualization remains essential [24,25]. This aligns with our findings and recent literature highlighting the consequences of inaccurately diagnosing advanced T3 and T4 RCC and its high-risk features.

Open surgery is the standard for IVC thrombectomy but carries risks, particularly in cases requiring cardiopulmonary bypass or caval resection [25]. Limited literature explores the intraoperative IVC involvement discovery. One study found a 7.7% rate of unexpected IVC wall invasion not detected preoperatively, highlighting imaging limitations [23]. Despite the predictive prowess of MRI enhancement and IVC diameter, the need for more detail prompts the consideration of alternative modalities such as transesophageal echocardiography and intraoperative exploration, potentially amplifying the rate of UCO [23].

Our study has several limitations. First, the lack of clinical staging may have influenced UCO rates. Further sub-analysis on the upstaging of UCO could provide insights, as open approaches are typically preferred for higher-stage urological cancers. UCO and longer operating times may correlate with the upstaging of RCC due to unexpected renal or IVC involvement. Additionally, the absence of tumor thrombus classification in our NSQIP data limits our understanding of UCO-related vessel invasion. Similarly, laterality may influence surgical complexity due to anatomical differences between left- and right-sided tumors, particularly in the context of vascular involvement such as tumor thrombus. The lack of data on individual surgeon decision-making when opting for UCO, as subjective factors such as experience, preference, and training, can also influence the choice for UCO. We anticipate that as robotic surgery adoption increases, the rates of robotic nephrectomy with tumor thrombectomy will increase. Lastly, while we included both laparoscopic and robotic-assisted procedures under the broader category of minimally invasive surgery, we acknowledge that there are distinct technical differences between these approaches that may affect the likelihood of conversion. Although we included surgical approach as a covariate in our regression model, we recognize that finer distinctions between robotic and conventional laparoscopy may independently contribute to outcomes. Finally, advanced imaging technologies and intraoperative assessment methods may further enhance this analysis in the future.

## Conclusions

This study underscores the critical role of advanced tumor stage, systemic disease burden, and anatomical complexity in driving unplanned conversion to open during minimally invasive nephrectomy for RCC. Future research should aim to refine preoperative risk stratification by addressing selection biases, incorporating clinical staging data, and expanding datasets to include cases involving IVC thrombus. Larger prospective studies are needed to compare outcomes between minimally invasive and open approaches for IVC thrombectomy, particularly as they relate to intraoperative decision-making and long-term oncologic safety. Moreover, investigating the timing, indications, and intraoperative triggers for conversion may offer valuable insights into perioperative risk management and optimize surgeon-patient communication. Enhanced predictive tools, including advanced imaging modalities and intraoperative assessment strategies, may improve preoperative planning and reduce the incidence of unanticipated conversions, ultimately contributing to more individualized and safer surgical care.

## Appendices

Table 5 shows CPT codes for partial and radical nephrectomy procedures.

**Table 5:** CPT Codes for Partial and Radical Nephrectomy Procedures CPT: Current Procedural Terminology

Procedure Type	CPT Code	CPT Description
Partial nephrectomy	50543	Laparoscopy, surgical; partial nephrectomy
	50240	Nephrectomy, partial
Radical nephrectomy	50546	Laparoscopy, nephrectomy with partial ureterectomy
	50548	Laparoscopy, nephrectomy with total ureterectomy
	50545	Laparoscopy, radical nephrectomy
	50236	Nephrectomy with total ureterectomy and bladder cuff, separate incision
	50230	Nephrectomy with partial ureterectomy, open rib resection, radical
	50225	Nephrectomy with partial ureterectomy, open rib resection, complicated
	50220	Nephrectomy with partial ureterectomy with open rib resection
	50234	Nephrectomy with total ureterectomy and bladder cuff, same incision
